# Differences in utility scores obtained through Brazilian and UK value sets: a cross-sectional study

**DOI:** 10.1186/s12955-015-0318-1

**Published:** 2015-08-06

**Authors:** Maíra Libertad Soligo Takemoto, Nilceia Lopes da Silva, Ana Carolina Padula Ribeiro-Pereira, Arthur Orlando Correa Schilithz, Cibele Suzuki

**Affiliations:** ANOVA – Avaliação de Tecnologia em Saúde, Rua da Ajuda, 35, sala 704 – 7° andar Centro, Rio de Janeiro, RJ Brazil; Novartis Biociências S.A., Avenida Prof. Vicente Rao, 90, São Paulo, SP Brazil

## Abstract

**Background:**

Multiple sclerosis (MS) is a chronic disease associated with several impacts; especially regarding patients’ health-related quality of life (HRQL). EuroQol 5 Dimensions questionnaire (EQ-5D) provides self-reported analysis of HRQL and utility scores. Although the British algorithm to convert EQ-5D responses into utility is the most used in the literature, national settings is more appropriate for health policy decision makers. A Brazilian algorithm is available, but not used in MS patients yet. Primarily, this study aimed to address potential differences in utility scores obtained through Brazilian and British value sets. Secondary objective was to determine the role of disability, fatigue and patients socio-demographic and clinical characteristics relevant to MS on the utility scores reported by Brazilian patients.

**Methods:**

Cross-sectional study with MS patients treated in 8 Brazilian sites. Patients were interviewed about socio-demographic and clinical characteristics, self-reported disability level, HRQL and impact of fatigue on daily living. Disability level, HRQL and impact of fatigue were assessed using the Expanded Disability Status Scale (EDSS) and the Brazilian versions of EQ-5D-3L and Modified Fatigue Impact Scale (MFIS-BR), respectively. Patients were classified in subgroups according to EDSS (mild: 0–3; moderate: 4–6.5; severe: >7) and the self-perceived impact of fatigue (absent: ≤38 points; low: 39–58; high: ≥59). EQ-5D-3 L data was converted into a utility index using an algorithm developed by a Brazilian research group (QALY Brazil) and also the UK algorithm. Differences between utility scores were analysed through Wilcoxon test.

**Results:**

Two hundred and ten patients were included in the study. Utility index mean scores of 0.59 (SD = 0.22) and 0.56 (SD = 0.32) for the Brazilian and UK algorithms were observed, respectively, without statistically significant difference for the distribution of data (*p* = 0.586). However, when utility scores were lower than 0.5, Brazilian algorithm provided higher estimates than UK with a better agreement between the scores found closer to 1. The same trend was observed when data was stratified for EDSS and impact of fatigue, with statistically significant difference between scores in categories of mild/severe disabilities and absent/high impact of fatigue.

**Conclusions:**

Results suggest that Brazilian value set provided higher utility scores than the UK, particularly for measures below 0.5.

## Background

Multiple sclerosis (MS) is an inflammatory and demyelinating disease of the central nervous system, which in most cases involves motor, sensory, visual and cognitive alterations, besides other clinical manifestations [1, 2]. It is estimated that about 2.5 million people are living with the disease worldwide. In Brazil, the estimated prevalence ranges from 1.36 to 18.1/100,000 inhabitants, depending on the characteristics of the studied population [1, 3].

The EQ-5D-3L is widely used to measure health-related quality of life in MS. It allows both the descriptive assessment of self-reported impairment in generic dimensions of health and the estimation of utility scores, being one of the most employed instrument in burden of illness studies across several therapeutic areas [4–17]. Most of the studies using EQ-5D-3L to calculate utility scores in MS patients use the algorithm developed for the United Kingdom (UK), however a national value set is more appropriate for health policy decision makers [18, 19]. Recently, an algorithm to estimate Brazilian preference weights for the 243 health states was described by a Brazilian research group (QALY Brazil), which conducted a household survey using the time trade-off technique to value EQ-5D-3L health states [20].

Thus, the primary aim of this study was to address potential differences in utility scores obtained through Brazilian and British value sets. Additionally, the secondary objective was to determine the role of disability, fatigue and patients socio-demographic and clinical characteristics relevant to MS natural history on the utility scores reported by Brazilian patients.

## Methods

### Study design and patient assessment

This was a multicenter, cross-sectional study conducted in eight centers in Southern and Southeastern Brazilian regions, specialized in MS diagnosis and treatment. Patients were screened for eligibility and invited to participate consecutively, as they attended a routine visit at study sites. If they agreed, they were asked to sign an informed consent. Patients were deemed eligible if they were at least 18 years old and if they had clinical diagnosis of MS according to the revised McDonald criteria [21]. Patients were excluded if they had any physical or mental condition that would impair their understanding and ability to answer the study interview (particularly the self-reported measures of HRQL and fatigue) and/or they were already enrolled in a clinical trial at the time of enrollment. This study was approved by the independent Ethics Committees of each participating center.

During data collection, which occurred between November/2011 and May/2012, patients answered a face-to-face structured interview conducted by a clinical research assistant during an outpatient routine visit, in order to collect self-reported variables about socio-demographics and clinical aspects, disability level, health-related quality of life (HRQL) and impact of fatigue on daily living.

### Disability level

Disability level was assessed by using the self-reported Expanded Disability Status Scale (EDSS), a well established method to assess MS-related disability in both clinical trials and epidemiological studies [22]. Patients were classified as having mild, moderate or severe disability according to the following cutoffs: 0–3 (mild), 4–6.5 (moderate), and ≥7 (severe), as previously described by other authors [4–6].

### Health-related quality of life

EQ-5D-3L was used to assess health-related quality of life. This instrument measures generic quality of life through five different domains (mobility, self-care, usual activities, pain/discomfort and anxiety/depression) in which patients choose between 3 response levels (no problem, some problem and serious problem) [23].

### Utility

Data obtained from EQ-5D-3L was used to calculate an index -utility score- which ranged from 0 to 1 (where death = 0 and perfect health = 1). Utility measures are based on patient judgment and indicate preferences for health states, which means how well each state is preferred by individuals, groups or society [24].

Patient self-reported health status (consisting of the answers to each EQ-5D-3L domain) was converted into the EQ-5D-3L index using the UK value set, as described by Dolan et al. (1997) and also using the algorithm developed by QALY Brazil Group in a Brazilian population-based study [18–20].

### Fatigue

Fatigue was assessed through MFIS-BR (Modified Fatigue Impact Scale, Brazilian Portuguese version) which measures the impact of fatigue on quality of life through 21 questions on physical, cognitive and psychosocial domains [25]. Impact of fatigue was considered as absent when total score was ≤38 points, low when the score was between 39 and 58 points and high when ≥59 points. For the purposes of this study, the impact of fatigue was used as an independent variable potentially associated with the utility scores estimates.

### Statistical analysis

Shapiro-Wilk and Jarque-Bera tests were used to test normal distribution of data. All data were submitted to exploratory analysis to describe measures of central tendency and dispersion for continuous variables, and frequency measures for categorical variables. Non-parametric tests were employed in the analysis due to the non-normal distribution of data. For comparison of means among patient subgroups (according to MS-relevant demographic and clinical characteristics), *t*-test and Kruskal-Wallis were used. Wilcoxon for paired samples was used to test the difference between UK and Brazilian scores. Intra-class correlation coefficient (ICC) was used to assess the reliability between measures of utility. Bland–Altman plot were also constructed to further assess agreement between BR and UK values, as previously described [26]. Analyses were performed using the statistical software STATA (version MP12; StataCorp. 2011; College Station, TX). The p-value ≤0.05 was assumed for statistical significance.

### Ethical approval and consent

All procedures performed in studies involving human participants were in accordance with the ethical standards of the institutional and/or national research committee and with the 1964 Helsinki declaration and its later amendments or comparable ethical standards. Informed consent was obtained from all individual participants included in the study. The study protocol was submitted and approved by each Institutional Review Board of the eight participating sites: Centro de Pesquisa Clínica do HCPA (Protocol number: 110267); Hospital de Clínicas da UNICAMP (Protocol number: 433/2011); Santa Casa de São Paulo (Protocol number: 173/11); Hospital das Clínicas da Faculdade de Medicina da USP (Protocol number: 0571/11); Centro de Pesquisas da Neurologia da UNIFESP (Protocol number: 0769/11); Universidade Metropolitana de Santos – UNIMES (Protocol number: 018/2011); Centro de Pesquisas Clínicas do HSL – PUCRS (Protocol number: 12/05750); and Clínica Neurológica e Neurocirúrgica de Joinville Ltda (Protocol number: 12004).

## Results

### Patient demographic and disease characteristics

Two hundred and ten consecutive patients met eligibility criteria and were included in the study. Patients’ demographic and disease characteristics are shown in Table 1.

**Table 1:** Clinical and demographic characteristics of the sample (N = 210)

	N (%)
Age (years)	
Mean[SD]	40.7 (11.5)
Median[IQR]	40 (31–49)
Min-Max	18 – 71
Gender	
Male	62 (29)
Female	148 (70)
Living	
Alone	11 (5)
Family	199 (95)
Educational level	
Never been to school	-
Elementary school (complete/incomplete)	35 (16)
High school (complete/incomplete)	100 (48)
Graduation (complete/incomplete)	61 (29)
Post-Graduation (complete/incomplete)	13 (6)
No information	1 (1)
Occupation	
Employed	68 (33)
Not employed^a^	140 (67)
MS type	
Relapsing-remitting	166 (79)
Secondary progressive	44 (21)
Diagnosis time (years)	
Mean[SD]	7.9 (6.2)
Median[IQR]	6 (3–11)
Min-Max	1 – 30
Disability (EDSS)	208 (99)
0 - 3	84 (40)
4 - 6.5	91 (44)
7 - 9	33 (16)
Impact of fatigue (MFIS-BR)	210 (100)
Absent	102 (49)
Low	67 (32)
High	41 (19)
Recurrences	102 (49)
Number of recurrences in the last year per patient with at least one episode	
Mean[SD]	1.6 (1.0)
Median[IQR]	1 (1–2)
Min-Max	0 – 5

^a^Not employed includes students, housewives, retirees and unemployed

### Utility

**Fig. 1:**
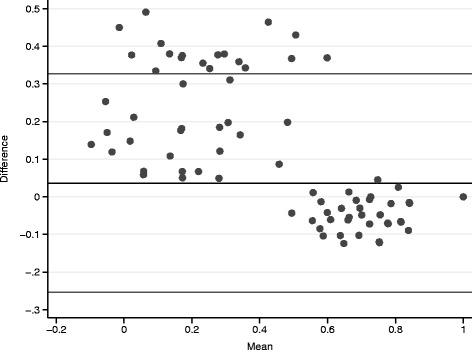
Bland–Altman plot - comparison of utility scores derived by Brazilian and UK algorithms. Difference between the two utility scores (y-axis) is plotted against their mean (x-axis)

Mean utility scores of 0.59 (SD = 0.22) and 0.56 (SD = 0.32) for the Brazilian and UK algorithms were observed for the total sample, with no statistically significant difference among the distribution of data (*p* = 0.586, Wilcoxon test for paired samples). Although statistical significance was not reached, the Bland-Altman plot depicted in Fig. 1 demonstrates that when utilities scores are lower than 0.5, Brazilian algorithm provides higher estimates than UK, and that a better agreement between estimated utility scores is found closer to 1. A good correlation among the estimates was found (ICC = 0.92), despite differences in distribution of data, which may explain the lack of statistical significance in the comparison analyses.

#### Utility scores and patients characteristics

Comparison of utility scores derived by Brazilian and UK algorithms according to socio-demographic and clinical characteristics is shown in Table 2. Subgroups segmented by age, educational level, type of MS and duration of the disease demonstrated statistically significant differences when considering both Brazilian and UK algorithms. In both algorithms (Brazilian and UK), the utility score increased in accordance with the increase in educational level (p < 0.001) and the disease duration (*p* = 0.002). When age groups were compared, also in both algorithms, those with age between 31 and 60 years old had lower utility scores (*p* = 0.004). Higher utility scores were observed for relapsing-remitting MS patients, when compared to those with secondary progressive MS with both algorithms (p < 0.001).

**Table 2:** Utility measures according to socio-demographic and clinical characteristics

Dimension	BR utility mean (SD) *p-value*	UK utility mean (SD) *p-value*
Age		
18-30	0.675 (0.20)	0.679 (0.24)
31-60	0.564 (0.23)	0.509 (0.34)
61+	0.599 (0.22)	0.633 (0.32)
	0.004	0.004
Gender		
Male	0.561 (0.21)	0.539 (0.29)
Female	0.607 (0.23)	0.566 (0.33)
	0.126	0.320
Living		
Alone	0.524 (0.15)	0.525 (0.26)
Family	0.597 (0.23)	0.558 (0.32)
	0.152	0.364
Educational level		
Until elementary school	0.481 (0.23)	0.410 (0.35)
Between elementary and high school	0.582 (0.22)	0.531 (0.32)
Graduation and Post-graduation	0.663 (0.19)	0.667 (0.26)
	<0.001	<0.001
Occupation		
Not employed	0.536 (0.22)	0.492 (0.33)
Employed	0.709 (0.18)	0.693 (0.24)
	<0.001	<0.001
MS Type		
Relapsing-remitting	0.635 (0.21)	0.606 (0.30)
Secondary progressive	0.440 (0.21)	0.380 (0.33)
	<0.001	<0.001
Recurrence		
Yes	0.579 (0.21)	0.535 (0.31)
No	0.606 (0.23)	0.576 (0.33)
	0.249	0.204
Diagnosis time		
<10 years	0.534 (0.24)	0.463 (0.35)
≥10 years	0.635 (0.20)	0.622 (0.27)
	0.002	0.002
Total		
Mean(SD)	0.593 (0.223)	0.557 (0.319)
Median(IQR)	0.625 (0.472 - 0.742)	0.656 (0.414 - 0.779)

#### Utility scores and EDSS

**Fig. 2:**
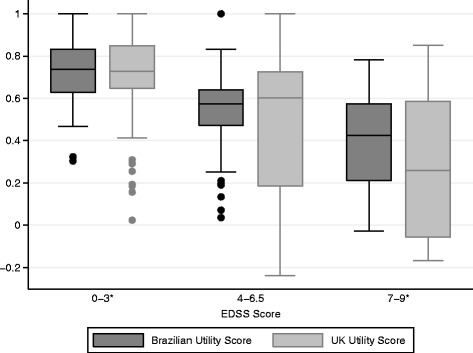
Brazilian and UK utility scores distribution stratified by EDSS levels. Medians of utility scores according to the three groups of disabilities (mild, moderate and severe) in MS patients. *Significant in the Wilcoxon test for paired samples

**Fig. 3:**
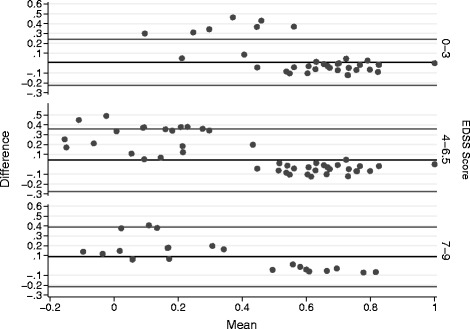
Bland-Altman plot: Brazilian and UK utility scores stratified by EDSS levels. Difference between the two utility scores (y-axis) is plotted against their mean (x-axis), according to the three groups of disabilities (mild, moderate and severe) in MS patients

Utility measures were also assessed by disability levels (EDSS) and the distribution of data is presented in box plots shown in Fig. 2. It was observed that the utility score decreased in accordance with the increase of disability level (p < 0.001 for both Brazilian and UK values). Patients with mild symptoms of disability (EDSS: 0–3) had a mean utility score of 0.738 (SD = 0.17) and 0.731 (SD = 0.21) for Brazilian and UK algorithms, respectively. When the Wilcoxon analysis was performed, a significant difference among the distribution of mean scores was observed (*p* = 0.007). The existence of outlier values in the UK analyses shown in Fig. 2 may justify the difference found although the mean values were very similar. Data shown in the Bland-Altman plot corroborates these findings, describing an agreement for measures above 0.5 and higher values for Brazilian measure when the score is below this cut-off (Fig. 3).

When the group with severe disabilities (EDSS: 7–9) was assessed, mean utility values observed were 0.387 (SD = 0.22) with the Brazilian algorithm and 0.299 (SD = 0.34) with the UK algorithm. Among these patients, a significant difference between the distribution of measures was also observed (*p* = 0.013, Wilcoxon test) – the score calculated by the UK value set showed a greater range than the Brazilian estimate (Fig. 2). Following the same trend described by patients with mild disability, an agreement is observed for higher utility values and the Brazilian measure tended to be higher when the score is below 0.4 (Fig. 3).

The only group in which a statistical significant difference among the utility measures was not observed was for patients with moderate disability (EDSS: 4–6.5) (*p* = 0.917), with mean scores of 0.533 (SD = 0.18) and 0.492 (SD = 0.30) for Brazilian and UK algorithms, respectively (Fig. 2). Despite the absence of a significant difference, the same pattern was observed in the Bland-Altman plot for this group, where a disagreement is found when the score is below 0.4 (Fig. 3).

#### Utility scores and fatigue

**Fig. 4:**
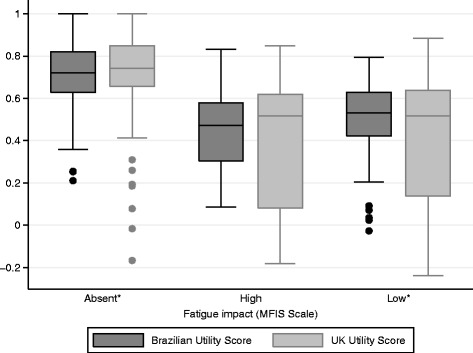
Brazilian and UK utility scores distribution stratified by fatigue levels. Medians of utility scores according to the three levels of fatigue impact (absent, high and low) in MS patients. *Significant in the Wilcoxon test for paired samples

Utility measures were then stratified by fatigue levels (Fig. 4). There were significant differences between the three MFIS-BR subgroups, showing an apparent association between the impact of fatigue and the utility score (p < 0.001 for both Brazilian and UK measures).

**Fig. 5:**
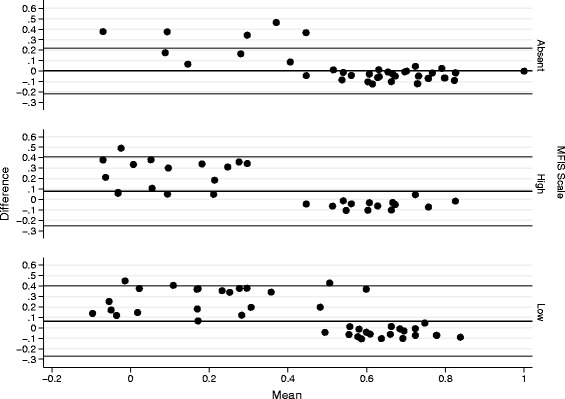
Bland-Altman plot: Brazilian and UK utility scores stratified by fatigue levels. Difference between the two utility scores (y-axis) is plotted against their mean (x-axis), according to the three levels of fatigue impact (absent, high and low) in MS patients

Patients with no fatigue impact (MFIS-BR: <38) had a mean utility score of 0.719 (SD = 0.18) and 0.718 (SD = 0.24) using Brazilian and UK algorithms, respectively (*p* = 0.001, Wilcoxon test for differences in data distribution). Wilcoxon analysis evaluates the distribution of data rather than the difference of means; therefore, the outlier values observed in UK measure may explain the difference found, even with very similar mean values (Fig. 4). The same trend was observed in Bland-Altman plot, where a difference between utility scores and fatigue impact was observed for a utility score below 0.5 (Fig. 5).

Among patients with high fatigue impact (MFIS-BR: ≥59), mean utility observed was 0.448 (SD = 0.20) and 0.368 (SD = 0.31) for Brazilian and UK algorithms, respectively. Comparing the distribution of the scores, significant differences were found (*p* = 0.027) and the measure provided by the UK algorithm had a greater range (Fig. 4). Similar to the pattern observed above, an agreement is apparent only for higher utility values (above 0.4) (Fig. 3).

Patients with low impact of fatigue (MFIS-BR: 39–58) had a mean utility score of 0.488 (SD = 0.20) and 0.424 (SD = 0.31) for Brazilian and UK algorithm, respectively. No differences in distribution of data was observed in this group (*p* = 0.233), however the same pattern of a higher difference among the two estimates was observed when the utility value is lower than 0.5 (Figs. 4 and 5).

## Discussion

This study aimed to address potential differences in utilities derived from the well-established UK value set, as described by Dolan et al. [18], and the newly published Brazilian value set, obtained through a household-based study conducted with 9,148 subjects in Minas Gerais state and Rio de Janeiro, Porto Alegre and Recife cities [27]. Patients’ health status was assessed by using EQ-5D-3L and then the EQ-5D-3L data were converted into a utility index using Brazilian and UK value sets. To our knowledge, this is the first study that used the algorithm proposed by QALY Brazil group in a Brazilian sample of patients with MS and also compared the findings with the most used method in literature.

Patients participating in the study were mainly female with a mean age of 40.7 years old. Demographic characteristics are comparable with those previously described for Brazilian MS patients and in studies that assessed quality of life in MS worldwide [4–16, 28–31]. Most of the patients had relapsing-remitting MS, moderate disability and a mean utility score of 0.59 (SD = 0.22) and 0.56 (SD = 0.32) for the Brazilian and UK algorithms, respectively. Other studies with similar clinical characteristics described utility scores ranging from 0.491 to 0.698 in MS patients [11, 13, 30].

Considering the total sample, statistical significant differences among the Brazilian (0.59 [SD = 0.22]) and UK (0.56 [SD = 0.32]) algorithms were not observed (*p* = 0.586, Wilcoxon test for paired samples). This finding is different compared to results from studies comparing value sets for Argentina [32], Chile [32], Denmark [33], Japan [26], United States [26, 33, 34], UK [26, 32–35] and Spain [35]. However, similar to the results described here, all studies so far have shown lower values when UK algorithm was used for analysis (as compared to the local value set). Statistical tests comparing distribution of data showed that most differences between algorithms can be observed at lower utility scores as shown in this study and also in previous studies comparing local value sets with the one from UK [26, 32–34].

Differences among utility scores have been attributed in the literature to two main factors: methods used to collect and to rate each of the EQ-5D-3L health status; and cultural characteristics of the sample used [18, 27]. The most important differences among the methods used for UK and the QALY Brazil group were the number of health states used to estimate the value sets and modifications in the data collection process, both proposed by Kind (2009). However, the method to value each of the health states was the same (the time-trade-off technique) [36]. The EQ-5D-3L questionnaire provides 243 possible health states and valuation studies employ a subset of those health states and then apply statistical modelling to derive the remaining states. The Brazilian valuation study used 99 health states while the UK used 42 health states [27, 37]. The use of greater than 42 health states in the rating process was described only by the Brazilian and South Korean studies and researchers have discussed that it may provide the most simple and robust models [38–47]. The protocol proposed by Kind [36] brings three main updates to the EQ-5D-3L health states valuation process, which consists in shuffling cards describing the states before patients classify each one, the exclusion of the “unconscious” health state and the procedure of giving all cards at the same time to subjects. The rating of value sets is based on the time trade off method, where patients determine how long they could live under the proposed health state and whether it seems similar to death or perfect health. Cultural characteristics may influence the final model of the developed algorithm. To investigate potential cultural factors that may influence the difference in utility scores is not the scope of the present analysis, but previous authors have suggested that this may be explained by country-specific differences in the way people perceive and value health conditions [26, 32, 33, 35].

This study also assessed the role of disability (according to EDSS disability level), fatigue (using MFIS-BR) and patient’s socio-demographic and clinical characteristics relevant to MS natural history on the utility scores reported by Brazilian patients. In terms of self-reported EDSS subgroups (0–3, 4–6.5, 7–9), the increase in self-perceived disability level was accompanied by a decrease in the utility index for both Brazilian and UK value set, which are similar with findings from previous studies [4–16, 30, 31, 48–50]. Regarding the assessment of self-reported impact of fatigue, the results observed in our study using the MFIS-BR (59 %) differed from data previously described for Brazil. Nogueira et al. (2009) found higher frequencies of self-reported impact of fatigue (69 %, using the MFIS-BR) and Mendes et al. (2000) using the Fatigue Severity Scale reported a frequency of 67.4 % [51, 52]. Despite this fact, an association between utility and fatigue was also observed, as previously described by other authors who examined the same association using different quality of life measures [52]. Other variables such as age, educational level, employment status, MS type and disease duration were also significantly associated with utility scores. Those between-groups differences were consistent for both Brazilian and UK values.

It is important to consider that this study presented some limitations. Although this was a multicenter study, all study sites were from South and Southeastern Brazilian regions, which are different from other regions in terms of socio-demographic characteristics; and in terms of coverage and access to health care services. Thus, findings may not be representative from the entire country. Another limitation of this study was the self-reported approach to the data collection process, which can lead to memory bias – but is the most adopted approach in patient-reported outcomes studies due to the nature of targeted data. Regarding the variables assessed in this analysis, clinical characteristics (type of MS, recurrence and disease duration) are probably the most prone to bias if self-reported. Thus, the association between those variables and utility scores in MS can be further addressed in studies using other source of data or even combining different ones.

In spite of that, considering the widespread use of EQ-5D-3L in the decision process for evaluating new therapies in health systems worldwide, through cost-utility analysis, these findings could markedly be relevant for policy makers during the health technology assessment of MS treatments that can affect patient’s quality of life by slowing disability worsening and postponing progression to secondary progressive MS, reducing fatigue symptoms, and favoring work productivity [53].

## Conclusions

The results suggest that the Brazilian value set provides higher EQ-5D-3L index scores than the UK, particularly for utility scores below 0.5 (the lower the utility, the higher the discrepancy among valuation methods). However, the impact of the differences in these EQ-5D-3L index scores on the outcome of cost-utility analysis needs to be further addressed.
